# In-depth comparative analysis of *Tritrichomonas foetus* transcriptomics reveals novel genes linked with adaptation to feline host

**DOI:** 10.1038/s41598-022-14310-x

**Published:** 2022-06-16

**Authors:** Andrés M. Alonso, Nicolás Schcolnicov, Luis Diambra, Veronica M. Cóceres

**Affiliations:** 1 Laboratorio de Parasitología Molecular, Instituto Tecnológico Chascomús (INTECH), CONICET-UNSAM, Intendente Marino km 8.2, 7130 Chascomús, Provincia de Buenos Aires Argentina; 2 CREG, Universidad Nacional de La Plata-CONICET, La Plata, Argentina; 3 Laboratorio de Parásitos Anaerobios, Instituto Tecnológico de Chascomús (CONICET-UNSAM), Chascomús, Argentina; 4 Escuela de Bio y Nanotecnologías (UNSAM), Chascomús, Argentina

**Keywords:** Computational biology and bioinformatics, Microbiology

## Abstract

*Tritrichomonas foetus* is a flagellated parasite able to infect cattle, cats, and pigs. Despite its prevalence, feline tritrichomonosis has received markedly less attention than venereal infection, and little information about the molecular mechanisms that participate in feline host infection is available. Through a bioinformatics approach, we integrated public transcriptomic data for three *T. foetus* isolates and explored the differences at transcript level with a focus on pathogenesis and adaptation processes, particularly for the feline isolate. Our analysis revealed higher abundance levels of predicted virulence factors, such as proteases and surface antigens. Additionally, by a comparative and expression analysis of *T. foetus* genes, we proposed putative virulence factors that could be involved in feline infection. Finally, we identified a great proportion of predicted transcription factors of the MYB protein family and, by a promoter analysis, we revealed that MYB-related proteins could participate in the regulation of gene transcription in *T. foetus*. In conclusion, this integrated approach is a valuable resource for future studies of host–pathogen interactions and identifying new gene targets for improved feline tritrichomonosis diagnosis and treatment.

## Introduction

Parabasalia is a phylum of flagellated protozoans, being several of them important parasites in animals and human^1^. *T. foetus*, from class Tritrichomonadea and order Tritrichomonadida, has been described as a pathogen found in different animal hosts^2–5^. This protozoan parasite is an important venereal pathogen in cattle that causes endometritis, early embryonic death and temporary infertility in females. In contrast, *T. foetus* infects the ileum, caecum, colon, and is recognized as a primary cause of large bowel diarrhea in domestic cats^4^. Furthermore, *T. foetus* has been described as a pig parasite, being found in the nasal passages, stomach, caecum and colon of swine host^3,5,6^. While no morphological differences were documented that make distinguishable *T. foetus* isolates, molecular studies confirmed that *T. foetus* (bovine) and *T. suis* (porcine) belong to the same species, and are considered as synonymous^7^. In this context, a whole genome sequencing (WGS) study revealed remarkable differences between feline, bovine, and porcine isolates, but the data did not confirm that the feline isolate is a different species. Additionally, high-throughput technologies used for comparative transcriptomics and proteomics analysis revealed no molecular level divergence between the isolates, suggesting that feline, bovine, and porcine isolates may belong to the same species^8–13^. Taking into account that the genetic differences between *T. foetus* isolates are consistent but not sufficient to define the bovine, porcine and feline isolates as different species, we analyzed the differences among *T. foetus* isolates at the gene expression level, focusing on known pathogenic factors that would be related to the *T. foetus* pathogenic mechanism in the different hosts. Even though the pathogenic mechanisms of trichomonads are poorly understood, it has been suggested that dynamic conditions in the host's environment influence the trichomonas infection^14^. In this sense, differences have been reported in cysteine proteases expression profiles among the *T. foetus* isolates, which led to hypothesize an influence of niche/host context of parasite gene expression^12^.

In spite of its widespread prevalence and lack of effective therapies, feline *T. foetus* has received considerably less attention than venereal tritrichomonosis regarding the study of molecules related to pathogenesis and host interaction. In this context, it has been proposed that adherence to intestinal epithelium is dependent on a receptor-ligand interaction and has been reported that cysteine protease is related to the cytotoxicity of this parasite in the feline host^15,16^. Here, we integrated available *T. foetus* transcriptomics information by a bioinformatics analysis, in order to explore the differences among *T. foetus* isolates at the gene expression level, considering mainly the known molecular factors that could contribute to feline isolate pathogenesis.

Taking advantage of the genome draft assembly for *T. foetus* K1 strain^17^, we generated a guided transcriptome assembly for three publicly available transcriptomics pieces of data: PIG30/1 (pig), G10/1 (feline) and BP-4 (bovine) isolates. We determined the abundance of expressed transcripts confirming great similarities between BP-4 and PIG30/1 and marked differences in those isolates connected to G10/1. Using a comparative procedure, we found a set of pathogenic factors mainly expressed in G10/1 that were predicted to codify for BspA-like proteins, tetraspanin proteins, metalloproteases, papain-like proteases, calpain-like proteases, subtilisin-like proteases and Myb-like DNA binding proteins (MYB), these last known in other pathogens as key transcription factors. Finally, we revealed expression patterns for MYB proteins in G10/1 isolate, and by an *in-silico* promoter analysis, we observed the presence of DNA motifs in the sequences, suggesting that these transcription factors could be involved in gene transcriptional regulation in *T. foetus*.

## Methods

### Data acquisition and analysis

Raw data of RNA sequencing from *T. foetus* isolates: porcine (PIG30/1; SRX973684)^18^, bovine (BP4; SRX540117) and feline (G10/I; SRX540971)^11^, were obtained from sequence read archive database^19^. These data sets do not comprise biological replicas, which prevents differential analysis of gene expression. Quality check was performed by FASTQC tool^20^ and reads were filtered by trimmomatic software^21^. Surviving reads were aligned to *T. foetus* K1 reference genome (ASM183968v1) employing HISAT2 alignment tool^22^. Mapping reads for each sequence experiment were assembled, and transcripts abundance (FPKM) were calculated using cufflinks software^23^ (*T. foetus* K1 as reference). Resulting assemblies were merged to obtain a common transcriptome for the three sequencing samples by cuffmerge script^23^. A results inspection was performed by the cummeRbund package for R^24^. Transcripts with at least FPKM > 1 in one of the strains were kept and pseudocount = 1 were added to all the dataset for further exploratory analysis. Principal component analysis and scatterplots were constructed with R functions and customs scripts. For Venn Diagrams, transcripts with FPKM > 1 at each isolate were kept as independent ensembles for further comparison and construction of diagrams with R custom script. Supplementary Table S1 lists FPKM values for each transcript used in this work for the three isolates.

### Transcript annotation procedure

Transcript sequences (contigs) from the merged transcriptome were translated in the 6 reading frames by transeq tool^25^. The Longest translated sequences (100 aa) per transcript were kept and used as input on a massive annotation procedure using the interproscan tool^26^. Only pfam^27^, PANTHER models^28^ and ontology terms were kept for matching sequences. Total results from interproscan were filtered by expectation value (*E* *value* < 10^−3^), only the best match value was taken into account by a custom python script, results are presented in Supplementary Table S2. A custom python script was constructed to perform term count and Blast2go (v5.2) software^29^ was employed for Enzyme Commission numbers (EC) mapping; Hydrolases (EC: 3) mapping were analyzed by blastp using the curated MEROPS (v12.3) database of proteases^30,31^, results were filtered by *Evalue* < 10^−3^ (Supplementary Table S3).

### Virulence and pathogenic factors analysis

Results from annotation procedure (Supplementary Table S2) were filter by python script for calpain proteases (PF00648;PTHR10183), papain proteases (PF00112; PTHR12411; PTHR35899), GP63-like (PF01457;PTHR10942), subtilisin-like (PF00082), serine proteases (PF05577; PTHR11010), tetraspanin proteins (PF00335), BspA-like proteins (PF13306;PTHR45661), *Chlamydia* polymorphic membrane proteins (PF02415), MYB proteins (PF13921;PF00249;PTHR45614). Multiple sequence alignment was performed in Jalview software using MUSCLE v3.8.31 algorithm and secondary structure prediction was calculated by Jpred^32–34^. Signal peptide and transmembrane domains were predicted by SignalP v5.0 server and TMHMM Server v. 2.0 respectively^35,36^. *Treponema pallidum* Leucine rich repeat (TpLRR) pattern^37^, Lx(2)IxIx(3)Vx(2)IGx(2)AFx(2)Cx(2) was searched by python custom script allowing three mismatches. Spearman correlation coefficients for predicted virulence factors (FPKM values) were calculated and plotted on a heatmap by a custom R script.

### Agglomerative procedure and analysis

To reduce the redundancy of the data set, we performed a hierarchical clustering method (UPGMA) in order to group 26,927 transcripts in clusters by similar expression values. To estimate an adequate number of clusters, we performed the agglomerative procedure for different numbers of clusters and calculated a measure of the clustering merit (Davies-Bouldin index, DBI)^38^. Low values of DBI indicated good cluster structure; as a result we grouped 26,927 transcripts in 458 clusters (Supplementary Fig. S1). Cluster composition is listed in Supplementary Table S2 and the resulting cluster matrix (458 × 3) is shown in Supplementary Table S4. Heatmap and Word cloud plots were constructed by a Wolfram script, word weights were represented as log_2_ function and values higher than 2 were plotted.

### Promoter analysis

For the extraction of putative promoter sequence feature annotation of *T. foetus* K1 strain was downloaded from NCBI genome database (txid: 1,144,522). Upstream genomic regions, 20 base pairs, from each *T. foetus* K1 gene from translation initiation were extracted with bedtools suite^39^. Regions were scanned for patterns of *Trichomonas vaginalis* initiator of transcription (*Inr*) [TCA] CA [TCA] [TA] by a custom python script. Matching sequences were expanded to 300 base pairs upstream initiation of translation and the resulting sequences were scanned for motif M3 [AGT][AG]C[GC]G[TC]T[TAG], motif MRE-1/MRE-2r (TAACGATA), motif MRE-2f. (TATCGT)^31,32^ and vertebrates consensus MYB recognition element (MRE, [CT]AAC[GT]G)^33^ by a custom python script.

## Results

### Bovine, Porcine and Feline *T. foetus* transcriptomics overview

We conducted an integrative bioinformatics approach in order to find out whether the differences at gene expression level of pathogenic factors could be related to *T. foetus* adaptation to different hosts. First, we conducted a mapping experiment employing three available transcriptomics datasets for *T. foetus* strains: BP-4 (bovine), PIG30/1 (porcine) and G10/1 (feline)^11,18^. We used the available public genome for *T. foetus* (bovine K1 isolate)^17^ as reference. Our results demonstrated a high mappability rate of the three isolates against *T. foetus* K1 strain (Table 1), and thus, a close relationship among the different isolates at transcriptomic level with the bovine K1 reference isolate.

**Table 1:** *Tritrichomonas foetus* transcriptomes statistics.

	This work	PIG30/1^a^	BP-4^b^	G10/1^b^
Assembly size (nt)	51,862,250	47,094,268	37,882,427	29,525,551
Contigs (n°)	26,928	43,308	42,363	36,559
Largest	21,237	17,203	14,314	17,195
Shortest	71	201	201	201
Average	1744.38	1087	895.25	806.61
N50	2454	1503	1259	1178
Map versus K1 (%)	–	95.68	96.65	91.36

This work: Guided assembly performed in this work using *Tritrichomonas foetus* K1 genome as reference. Map versus K1 (%) refers to the mappability rate of isolate reads against reference used in this work. ^a^Summary of transcriptome statistics from Morin-Adeline et al.; 2015^18^, ^b^Summary of transcriptome statistics from Morin-Adeline et al.; 2014^11^.

Even though de novo* T. foetus* transcriptome assemblies were available^11,18^, it was documented that reference guided assembly presents advantages such as improving contigs length and redundancy, integrating annotation from genes in reference and the possibility of assembling transcripts of very low abundance, and predicting new genes that were not annotated on the reference genome^40^. According to this, we performed a guided transcriptome assembly (K1 isolate as reference) for the three isolates, and the three transcriptomes were merged to obtain a common assembly for the three isolates. Finally, we calculated the abundance values (FPKM) for each transcript in the merged transcriptome. As a result, we obtained a total of 29,361 transcripts (contigs), of which we kept those that had a value of FPKM > 1 on at least one of the isolates and we obtained a total of 26,927 transcripts for the three isolates. In Table 1 we showed differences between de novo transcriptome assemblies and our guided merged assembly. Next, we performed an annotation process over our transcriptome assembly by interproscan tool, as was described in the material and methods section, which resulted in 15,909 annotated transcripts (~ 59%).

**Figure 1:**
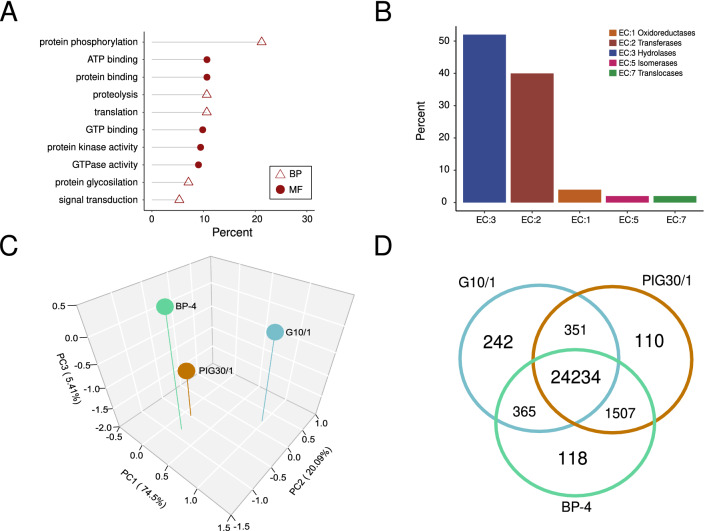
Exploring *Tritrichomonas foetus* transcriptome assembly. (**A**) Molecular functions and biological processes assigned to new assembled transcripts in this work; *Percent:* Counts of gene ontology terms represented as percent values for biological process or molecular functions; BP: Biological Processes, MF: Molecular Functions (**B**) Enzyme Commission number (EC) annotation; *Percent:* Counts of EC represented as percent values for each EC class; (**C**) Principal component analysis for the transcripts abundance (FPKM) data set. Colored points represent a dimensional reduction of transcript expression data for each isolate; (**D**) A Venn diagram that highlights differences and similarities between isolates at transcript abundance levels.

In our analysis we obtained 1181 transcripts whose homologous counterparts were assigned in the reference genome by the cufflinks algorithm. These new transcripts could be related to new genes. Next, we analyzed the biological processes and molecular function associated with new transcripts sequences. We obtained 102 mapped transcripts related to: (i) protein phosphorylation, proteolysis and translation process, (ii) functions like ATP, GTP binding and (iii) protein binding (Fig. 1A). As these results could be related to enzymatic roles, then we performed an Enzyme Commission number (EC) annotation process. From 49 annotated transcripts, we detected that the majority of the represented codes were related with Hydrolases (EC: 3), Transferases (EC:2), Oxidoreductases (EC:1), Isomerases (EC:5) and Translocases (EC:7; Fig. 1B). Since hydrolases are related to pathogenic processes and were the most represented in our analysis, we examined that group by searching homologous sequences in the MEROPS database. We were able to determine the relationship of these new genes with curated sequences of peptidases. We observed that transcript sequences (2) belonged to ubiquitin-specific protease family (family C19), one transcript sequence was included in the metallocarboxypeptidase family (family M14), and another sequence was included in the peptidase E family (family S51). Finally, we detected transcripts (2) related to cathepsin proteases (family C1). We also observed that these new proteases were expressed in the three *T. foetus* isolates, except one cathepsin that is not expressed by the G10/1 isolate (Supplementary Table S3). In this way, our annotation process contributed to finding new putative factors relevant for *T. foetus* biology.

### Abundance analysis of *T. foetus* transcripts

Next, we performed an exploratory analysis over our assembly aiming at revealing differences between isolates. Taking into account the abundance values from 26,927 transcripts, we explored the data set by a Principal Component Analysis (PCA) and we demonstrated that BP-4 and PIG30/1 isolates were grouped and distant from G10/1 isolate (Fig. 1C). In conclusion, the transcript abundance analysis revealed clear differences for G10/1 compared to the rest of the isolates, whilst BP-4 and PIG30/1 were similar. Since the pathogenic mechanisms of *T. foetus* feline isolate are poorly approached, we focused this work on exploring the differential abundance of transcripts in G10/1 isolate. By a closer inspection, we revealed that from 26,927 transcripts 24,234 were commonly expressed in three isolates, while 242 transcripts were only expressed in G10/1. Additionally, we observed that 118 and 110 transcripts were only detectable in BP-4 and PIG30/1 isolates, respectively (Fig. 1D). Since we are interested in finding differences at G10/1 isolate, we inspected those 242 only detectable transcripts in G10/1. Interestingly, within the transcripts that could be annotated, we detected that they were mostly related to putative MYBs (MYBs, (a total of 11 transcripts) and BspA-like proteins (5 transcripts). This observation was interesting since it has been widely documented in trichomonads and other protozoan that MYBs proteins are transcription factors that regulate the transcription of genes related to pathogenesis, cell differentiation and context adaptation^41,42^. Also, BspA-like proteins are known to increase adherence to host and improve adaptation to parasite niche^43^. Taking this into consideration, we performed a comparative functional analysis over the predicted amino acid sequences of the 11 *T. foetus* feline specifics MYBs (TfMybF) and the 5 predicted BspA-like proteins.

**Figure 2:**
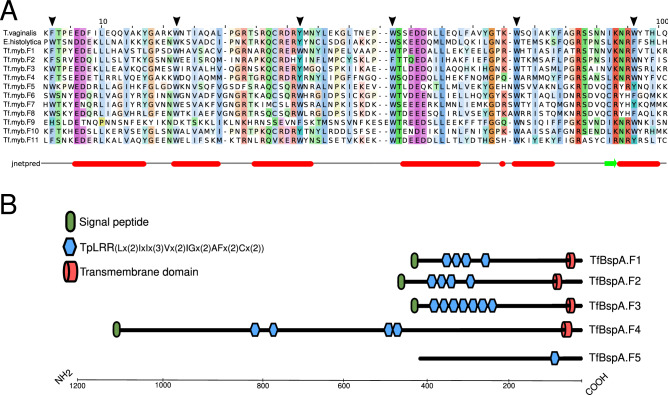
Specific abundant transcripts in G10/1 isolate are related to Myb-like and BspA-like proteins. (**A**) A multiple sequence alignment of predicted amino acid sequences for G10/1 MYB transcripts and *E. histolytica* (XP_648148.1) and *T. vaginalis* (XP_001307180.1); these are MYB validated proteins. Black arrowheads highlight conserved tryptophan (W) residues characteristic from the MYB family. At bottom, a schematic representation of second structure prediction by JPred algorithm, JNetPRED refers to the consensus prediction^34^. Red bars represent alpha helix and sheets are presented as green arrows; (**B**) Schematic description of predicted amino acid sequences for G10/1 isolate transcripts related to BspA-like proteins. TpLRR: *Treponema pallidum* leucine rich repeat^66^.

Typically, MYB proteins consist of one to four imperfect repeats (R) with three regularly spaced tryptophan (W) residues conserved. MYB proteins can also be divided into different classes depending on the number of adjacent repeats: R2R3-MYB, R1R2R3-MYB, 4R-MYB R1R2R2R1/2-MYB and MYB-related proteins (1R-MYB)^44^. Here, we performed a multiple sequence alignment and we obtained a group predicted MYBs proteins for feline isolate (G10/1), and then we confirmed their homology with the R2R3-MYBs proteins of *T. vaginalis* and *Entamoeba histolytica* (Fig. 2A). This finding revealed that *T. foetus* could be expressing functional proteins related to MYB transcription factors, since conserved elements necessary for protein function were detected in the amino acid sequences studied.

On the other hand, BspA-like proteins are known as surface antigens with a relevant role in host–pathogen interactions. These proteins are characterized by a specific type of leucine-rich repeats named TpLRR^43^. We subsequently analyzed the TpLRR motif in the 5 predicted amino acid sequences and observed that 4 of the predicted BspA-like proteins contained 4 to 8 repeats of the TpLRR motif (Fig. 2B). Signal peptides and transmembrane domains were detected in the majority of TvBspA protein sequences previously reported^45^. However, a predicted TfBspA showed only one TpLRR repeat and no signal peptide and transmembrane domain were predicted in this sequence; similar to BspA-like proteins described in *E. histolytica*^46^.

In conclusion, we predicted new putative virulence factors for *T. foetus* and we consider that the abundance of transcripts specific for genes encoding MYB proteins and BspA-like proteins observed in our analysis, could be suggesting a possible role of these genes in pathogenesis of *T. foetus* in feline host.

### Analysis of *T. foetus* G10/1 virulence and adaptation factors abundance

In order to analyze other factors related to pathogenesis that could be differentially abundant in G10/1 detected transcripts levels, we performed an extra comparative analysis. We analyzed G10/1 transcriptomics data by a comparative analysis of virulence and adaptation factors that were previously reported in trichomonads and other mucosal pathogen parasites such as: papain-like cysteine proteases and calpain-like cysteine proteases (cysteine proteases, CPs), GP63-like proteases (metalloproteinases), subtilisin-like proteases, serine proteases, tetraspanin proteins, BspA-like proteins, *Chlamydia* polymorphic membrane proteins (Pmp), and MYB transcription factors^45,47,48^. We observed a great distribution of MYB related transcripts in our assembly in addition to BspA-like and papain proteases related transcripts (Table 2). A total of 736 transcripts (Table 2, column 3, G10/1) were related to the factors proposed to analyze, and the most represented were MYBs, BspA-like, papain proteases and subtilisin-like proteases. The average abundance in G10/1 transcriptome was of 7.30 *log2* (FPKM), and the abundance of the pathogenic factors analyzed was higher than this average (except for BspA-like) (Table 2). The greatest abundance values were related to tetraspanins, which are surface antigens related to cell adhesion, migration, colonization and parasite aggregation in *T. vaginalis*^49^. Interestingly, Pmps family, despite having only three members, showed high values of abundance. This is an interesting result considering that it has been documented that Pmps in conjunction with BspA-like proteins could improve adherence in trichomonads^45^.

**Table 2:** Summary of the analyzed factors.

Factor	Domain ID	G10/1/Merged	Average abundance
Calpain proteases	PF00648;PTHR10183	8/9	8.19
Papain proteases	PF00112;PTHR12411;PTHR35899	61/64	7.47
GP63-like	PF01457;PTHR10942	13/14	8.03
Subtilisin-like	PF00082	38/40	7.85
Serine proteases	PF05577;PTHR11010	14/14	7
Tetraspanin proteins	PF00335	7/7	11.01
BspA-like	PF13306;PTHR45661	66/78	6.4
Pmp	PF02415	3/3	8.87
MYB	PF13921;PF00249;PTHR45614	526/552	8

Columns are composed as follows, (1) adaptative and virulence factors analyzed in this work; (2) identifiers corresponding to PFAM and PANTHER databases; (3) count number of transcripts in G10/1 transcriptome with FPKM > 1 and total factors identified in merged transcriptome; (4) *log*_2_ transformed value of FPKM average value for corresponding transcripts in G10/1 isolate.

**Figure 3:**
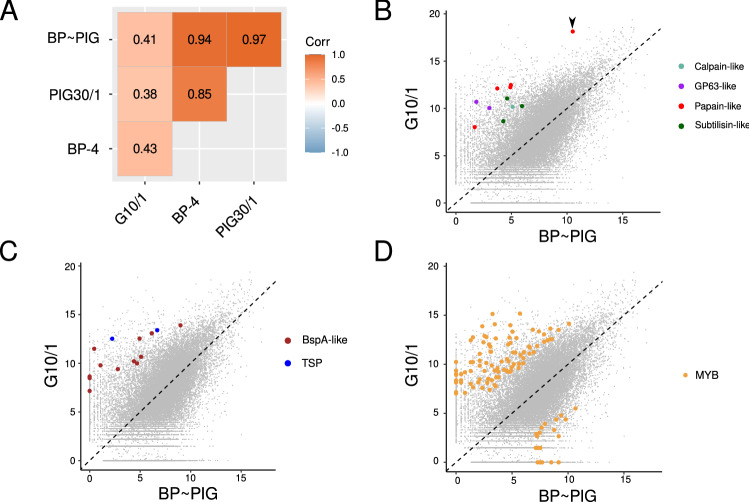
Analysis of predicted pathogenic and adaptive factors in the three isolates. (**A**) A correlation heatmap for the Spearman's coefficients for abundance values (FPKM) of predicted virulence factors analyzed in this work; Corr = Correlation coefficients code. Values are coded from light blue (− 1) to deep orange (1). Scatter plots for analyzed factors, those mostly abundant (fc > 4) in G10/1 isolate were highlighted (**B**) proteases; black arrow head: TfCP7 (**C**) surface antigens; TSP: tetraspanin proteins (**D**) MYB proteins; more expressed MYB transcripts (*log*_2_(*fc*) = 4) on BP ~ PIG are also shown.

It has been previously documented that cysteine proteases 7 (G10/1 isolate) and 8 (BP4 isolate) were differentially abundant in a comparative analysis^11^. Then, we analyzed the abundance of the factors previously studied in our analysis. As it can be observed in Fig. 3A, the abundance of the analyzed factors was mostly correlated between BP-4 and PIG30/1 isolates. In contrast, since we observed a positive correlation among isolates, coefficient values were lower when G10/1 is compared with the rest (*r* = 0,43 vs. BP-4 and *r* = 0,38 vs. PIG30/1). This observation revealed that PIG30/1 and BP-4 isolates could be expressing the set of analyzed factors at similar abundance levels in contrast to G10/1 isolate. In order to analyze differences between G10/1 and the rest of isolates in detail, we explored each group of factors separately.

Since bovine and porcine isolates were proposed as synonymous^7^ and given that our examination confirmed the strong correlation between both isolates, we will analyze the bovine and porcine isolates as a common isolate from here on. We calculated the average of abundance for each transcript of the analyzed factors generating a new merged data for BP-4 and PIG30/1 isolates (BP ~ PIG) that were used to compare them against the G10/1 isolate. Additionally, since publicly available data about the three isolates has no biological replicates to conduct a differential gene expression analysis, we calculated log_2_ fold change (*fc*) values for each transcripts for G10/1 versus BP ~ PIG. In order to show the differential expression of virulence factors we generated three scatter plots indicating those with high relative abundance on G10/1 isolate with respect to BP ~ PIG (*log*_2_(*fc*) > 4 and *log*_2_ (FPKM) > 7).

In Fig. 3B, we showed differentially abundant papain-like cysteine proteases of G10/1, and specifically the CP7 protease demonstrated differential expression, as it has been described previously^11^. Besides, we found four more CPs (papain-like) that could be expressed in G10/1 isolate that were homologous to TfCP1, crustapain, pro-cathepsin H and a hypothetical protein from K1 isolate. Furthermore, we detected two putative metalloproteinases homologous to the GP63 antigen from *Leishmania major* (also known as Leishmanolysin), two subtilisin-like proteases (serine proteases family) and one calpain from CPs family. When surface antigens were analyzed (Fig. 3C), we determined that 12 transcripts related to BspA-like proteins were differentially abundant in G10/1 isolate. Moreover, we detected two tetraspanin proteins related transcripts and no differential abundance for Pmp antigens was detected between G10/1 and BP ~ PIG. Finally, we observed a great proportion of transcripts related to the MYB protein family: a total of 92 in contrast to the 22 differentially abundant in BP ~ PIG; this observation could be related to an active and specific transcriptional process in G10/1 isolate (Fig. 3D).

### Pattern of transcript abundance in *T. foetus* G10/1 isolate

**Figure 4:**
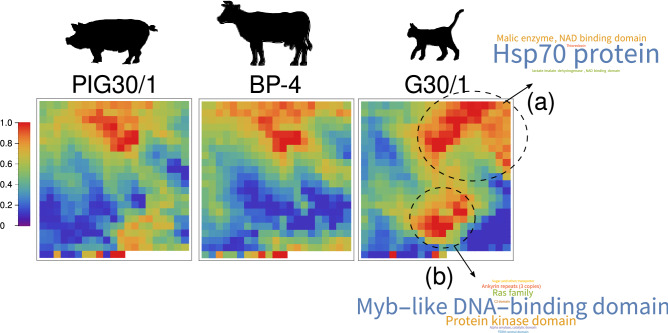
Clustering analysis reveals patterns of transcript abundance in G10/1 isolate. Transcript abundance data set was reduced to 458 variables or clusters of transcripts. Variables were represented in a heat-map of 22 × 21 cells. Abundance was represented by the average of each transcription in the cluster. Values were scaled between 0 and 1. Positions of the clusters in the heatmaps are the same for all isolates. WordClouds highlight the most represented annotated transcripts in that section of the heatmap. Distinctive regions for G10/1 isolate are highlighted as “(a)” and “(b)”.

Next, we evaluated if the differential abundance transcripts observed in G10/1 isolate were co-expressed with other transcripts that could be participating in similar processes (i.e. adherence or cytotoxicity), organized as characteristic patterns of expression for G10/1 isolate. To answer that question we conducted a more sophisticated analysis based on a clustering methodology. Since clustering processes are widely employed to reveal patterns in gene expression data sets^50^, we performed a reduction of dimensionality of the data by a hierarchical clustering process. A hierarchical clustering procedure allowed us to reduce the number of transcripts from 26,927 to 458 clusters of transcripts (or variables) for each strain. The results were represented on a heatmap of 22 × 21 clusters that were grouped by similar activity making more evident patterns in data. We observed similar patterns of clusters in PIG30/1 and BP-4 isolates, while we detected two regions of the heatmap that were characteristic for G10/1 isolate (Fig. 4). G10/1 heatmap analysis revealed the existence of an upper region of 20 clusters (Fig. 4, region “a”). In this section, the most represented sequences were annotated as Hsp70 family and malic enzymes, such as malate dehydrogenase and precursors of the ap65 adhesin. Furthermore, when we analyzed the clusters, we observed small amounts of factors previously described, such as subtilisin-like proteases, papain-like proteases, metalloproteinases and MYBs. Additionally, a great variety of transcripts related to glycolytic and oxidative processes were detected. Subsequently, we observed a region of 28 clusters composed principally by MYB related transcripts (Fig. 4, region “b”). Moreover, in region “b” we observed transcripts related to surface antigens (tetraspanin proteins and BspA-like proteins), Ras family proteins and transcripts related to phosphorylation processes.

Here, we revealed the existence of two differential regions observed only in G10/1 heatmap. We were also able to demonstrate that these regions showed enrichment of transcripts related to Hsp70 family, malic enzymes, like malate dehydrogenase and precursors of the ap65 adhesin and MYB related transcripts.

### Search for consensus MYB-DNA binding sites in *T. foetus* gene promoters

**Figure 5:**
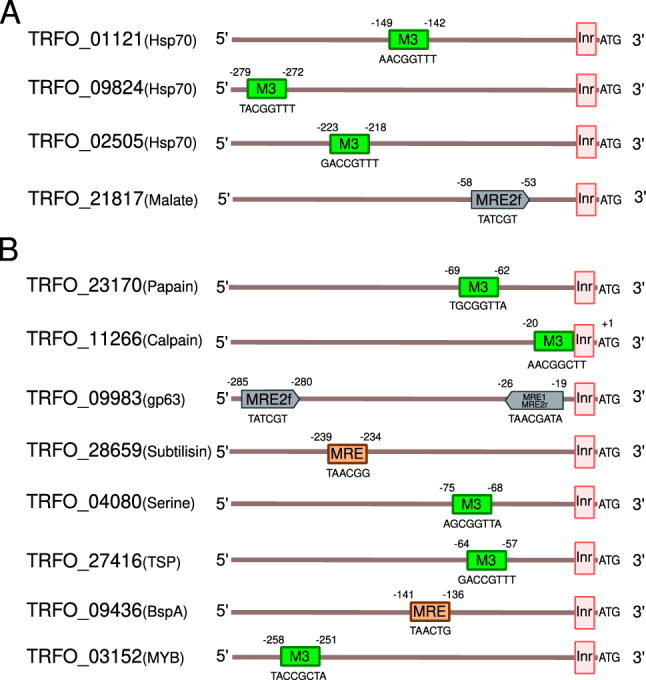
Predicted promoters from *Tritrichomonas foetus* genes with MYB binding motif. (**A**) A schematic representation of predicted promoters and the predicted MYB binding motifs from genes related to Hsp70 family and malic enzymes; (**B**) Representation of predicted promoters and the predicted MYB binding motifs for genes related to virulence and adaptive factors studies in this work.

Previously, MYB proteins were found to bind to promoter regions of Hsp70 family gene and malic enzyme genes, regulating transcriptionally the expression of this genes in protozoan parasites (*T. vaginalis* and *E. histolytica*)^47,51^. However, our hypothesis is that MYB DNA binding elements could be present in gene promoters of *T. foetus*, like Hsp70 or malic enzymes, since the great abundance of MYB transcripts observed in this work. Taking this into account, we performed an in silico analysis of consensus DNA binding motifs related to MYB transcription factors, with the purpose of determining if these characteristic DNA elements are conserved in *T. foetus.* Since no genome assembly for G10/1 or BP-4 (or PIG30/1) isolates are disposable at the date, we conducted our analysis over the reference genome (K1 strain). In order to predict the promoters of *T. foetus* genes (no core elements for *T. foetus* promoter were described at date), we decided to search promoter elements described in *T. vaginalis*, particularly the initiator of transcription (*Inr*); since heterologous expression of genes guided by *T. vaginalis* promoters in *T. foetus* were previously documented^52^. First, we obtained the 20 bp region upstream from initiation of translation for each gene annotated in the K1 assembly; next, a search was carried out considering the known *T. vaginalis* *Inr *pattern^53^. A total of 13,477 positive matching sequences were retrieved. For those matching sequences, we expanded the length from 20 to 300 bp upstream from the initiation of translation to search elements at short distance from the *Inr* since the reference was a draft assembly. Then, we searched sequence patterns related to the described MYB binding site as the consensus MYB recognition element from vertebrates (MRE) and the motif M3, motif MRE-1/MRE-2r and the motif MRE-2f. previously described in *T. vaginalis*^47,51,53^. We obtained 3957 predicted promoters that contained at least one of the mentioned binding sites (genes related to the predicted promoters are shown in Supplementary Table S5). Interestingly, we observed genes from the Hsp70 family (3) and malic enzyme (1) with MYB binding motifs in its predicted promoters. Figure 5A shows a schematic representation of the predicted promoters; the M3 element was matched in the analyzed region of genes related to the Hsp70 family. On the other hand, the analysis of the predicted promoter for the Malate dehydrogenase (TRFO_21817) revealed only a match for the MRE2f. element.

Finally, we expanded our analysis with the purpose of revealing MYB binding motifs in predicted promoters from virulence factors mentioned in this paper, since it was documented that MRE elements were predicted in *E. histolytica* BspA-like gene promoters^51^ (Table 3). In Fig. 5B we showed a schematic representation of the predicted promoters. Particularly, the M3 motif was the most represented among the factors studied here. While we got the first evidence of the existence of elements that resemble a transcriptional regulation in *T. foetus* by MYB related proteins, further studies are necessary to confirm this.

**Table 3:** Number of factors analyzed in this work with motifs in their predicted promoters.

Factor	Motif
Calpain proteases	2
Papain proteases	2
GP63-like	5
Subtilisin-like	3
Serine Proteases	1
Tetraspanin	2
BspA-like	6
MYB proteins	75

## Discussion

*Tritrichomonas foetus* is a relevant and efficient pathogen in the veterinary field, as it has the capability to colonize different hosts, such as bovine and feline, producing tritrichomonosis. It has also been reported as a parasite of porcine hosts *(T. suis*) and as an example of its great adaptability, it was documented as an opportunistic pathogen in humans^3,54^.

Previous studies on parasites of the same taxonomic order as *T. foetus* (Tritrichomonadida) revealed that genomic expression at different contexts could explain the adaptability and phenotype of isolates from the same species. In a transcriptomics study for virulent and attenuated isolates of *Histomonas meleagridis* (order Tritrichomonadida), specific transcripts for each isolate were identified^55^. Additionally, for *E. histolytica* and *T. vaginalis*, studies proposed that pathogenicity differences among isolates of the same protozoan parasite were due to changes in gene expression for virulence factors^14,56^. In fact, a transcriptomic analysis of *E. histolytica* demonstrated that changes in environmental conditions trigger the expression of virulence genes^57^, which could be related to an environmental influence on transcription regulation and pathogenesis.

In this work, we treated the BP-4 (bovine), PIG30/1 (porcine) and G10/1 (feline) isolates as part of the same species and we proposed differential abundant factors of G10/1 isolate that could contribute to the understanding of feline *T. foetus* pathogenesis. The available transcriptomics data for the three isolates was analyzed by an alternative approach to obtain a common transcriptome assembly for the three studied isolates, using bovine *T. foetus* K1 genome as reference, and then we performed the quantification of the assembly transcripts. Next, the assembly transcripts were annotated. Our analysis revealed clear differences at the expression level between G10/1 isolate and the other isolates (BP-4 and PIG30/1).

When transcriptomics of G10/1 were analyzed for pathogenic and virulent factors, we observed a particular abundance of proteases, surface antigens and, interestingly, transcription factors of the MYB family proteins; in fact, a differential abundance between isolates was observed. On the other hand, it is important to highlight that some transcripts were detected in G10/1 but not in BP-4 (or PIG30/1) isolate. This last observation could be related to the genetic differences observed between feline and bovine (or porcine) isolates, since these were predicted to have considerable impact on gene expression in *T. foetus* isolates^13^.

Specifically, we demonstrated the abundance and distribution of cysteine proteases: papain-like and calpain-like proteases, in addition to subtilisin-like proteases (serine protease family) and metalloproteases (GP63-like). Indeed, our analysis of differential abundance revealed the existence of five papain-like proteases (TfCP7 included), one calpain, two serine proteases and two metalloproteases (GP63-like), that were abundant in G10/1 isolate in comparison to BP-4 or PIG30/1 isolates. These results were according to previous reports in which the CPs and serine proteases were proposed as the principal virulence factors in feline *T. foetus*; although, metalloprotease activity was not detected in this analysis^16^. Moreover, differential CPs activity between feline and bovine isolates was documented^12^, which could be related to the differential abundance of CP proteases proposed in this work.

It has been reported that metalloproteases are secreted with papain-like proteases and subtilisin-like proteases, and have also been associated with *T. vaginalis* adherence to the host cell^58,59^. In this sense, a role of metalloproteases in virulence in other related intestinal parasites *E. histolytica* was also confirmed^60^. Thus, we do not rule out the role of GP63-like metalloproteases in feline *T. foetus* infection. Additionally, in *Trichomonas gallinarum*, GP63-like and subtilisin-like proteases were proposed as virulence determinants^61^. In this context, we suggested a potential role of cysteine proteases (papain and calpain), serine proteases (subtilisin-like proteases) and metalloproteases (GP63-like) as pathogenic factors and potential biomarkers for feline *T. foetus*.

Surface proteins mediate host–pathogen interactions and promote cell aggregations, thus expression profiles of three of these known protein families were analyzed in this work. Tetraspanin proteins (TSP) modulate adhesion, migration and proliferation and were confirmed as surface antigens for *T. vaginalis*^49,62^. Besides, BspA-like proteins mediate host pathogen interaction and were documented as relevant for pathobiology on *T. vaginalis* and *E. histolytica*^43,63^. Furthermore, it was documented that *Chlamydia* polymorphic protein membrane-like participates/collaborates with the BspA-like protein in improving the adherence of trichomonads to the host^45^.

In feline *T. foetus* we observed a great abundance for seven transcripts related to TSPs, and for three transcripts associated with Pmps. Furthermore, we observed a predicted *T. foetus* BspA-like family represented by 61 transcripts detected on G10/1 isolate. Although a relatively low abundance average was observed in the entire BspA-like family on G10/1, we demonstrated that the 12 members of BspA-like were differentially abundant in addition to TSPs (2) proteins. Differential abundance was not detected for Pmps. It is known that the surface antigens of *T. foetus* feline isolate could interact with the surface host proteins in a receptor-ligand manner^64–66^. In this sense, our results propose new parasite surface protein as targets of study in order to analyze the mechanism of parasite-host epithelium interaction.

Finally, we demonstrated that MYB transcription factors constitute an abundant family with 552 members, 92 of them were remarkably abundant in the G10/1 isolate, in contrast to 22 highly expressed in the merged isolate BP ~ PIG. These results are interesting findings since MYB proteins were documented in superior eukaryotes as regulating cell differentiation and stage conversion^67,68^. In pathogen parasites like *E. histolytica*, *Giardia lamblia*, *Toxoplasma gondii* and *Plasmodium*, MYBs proteins were documented as regulators of genes that were relevant for host adaptation and parasite cell differentiation^69–72^. Furthermore, in *T. vaginalis*, MYBs regulate transcription of adhesion proteins genes key for interaction with the host^73^. In this context, we can conclude that abundant expression of MYB proteins in G10/1 could be related to the plasticity of *T. foetus* and its capability to adapt to different hosts. In fact, our in silico promoter analysis reveals that MYB proteins could be regulating transcription of pathogenic genes of *T. foetus* as proteases (papain,GP63-like,calpain and subtilisin-like), surface antigens (TSPs and BspA-like proteins) heat shock proteins (Hsp70 and Hsp90-2 family) and MYB genes. Although these findings are coherent with the previously documented for *E. histolytica*^51^, further work is necessary to corroborate this hypothesis in *T. foetus*.

## Supplementary Information

Supplementary Information 1.

Supplementary Information 2.

Supplementary Information 3.

Supplementary Information 4.

Supplementary Information 5.

Supplementary Information 6.

## Data Availability

The datasets analyzed during the current study were obtained from Sequence Read Archive (https://www.ncbi.nlm.nih.gov/sra) database: porcine (PIG30/1; SRX973684), bovine (BP4; SRX540117) and feline (G10/I; SRX540971).

## Code availability

Principal scripts written for this work are available at 10.5281/zenodo.6371607. B. Bash script for extract promoters is available at https://github.com/RimGubaev/extract_promoters. All bioinformatics tools used in this work are listed in Supplementary Table S6.
