# Supplementary material for: In-depth comparative analysis of Tritrichomonas foetus transcriptomics reveals novel genes linked with adaptation to feline host
Source: Sci Rep. 2022 Jun 16;12:10057. doi: 10.1038/s41598-022-14310-x (PMC9203502; doi:10.1038/s41598-022-14310-x)
Supplement: Supplementary file 1 — Supplementary Information 1. [file 41598_2022_14310_MOESM1_ESM.docx]

**In-depth comparative analysis of *Tritrichomonas foetus* transcriptomics reveals novel genes linked with adaptation to feline host**

Andrés M. Alonso^a,d^*, Nicolás Schcolnicov^a,d^, Luis Diambra^b^, Veronica M. Cóceres^c,d^*

^a^Laboratorio de Parasitología Molecular, Instituto Tecnológico Chascomús (INTECH), CONICET-UNSAM, Chascomús, Argentina.

^b^ CREG,Universidad Nacional de La Plata-CONICET, La Plata, Argentina.

^c^Laboratorio de Parásitos Anaerobios, Instituto Tecnológico de Chascomús (CONICET-UNSAM).

^d^Escuela de Bio y Nanotecnologías (UNSAM)

**Legends to Supplementary Tables**

**Supplementary Table S1: Abundance values (FPKM) of transcripts in the assembly.** The abundance value is listed for 26928 transcripts at each isolate: PIG30/1, G10/1 and BP-4. BP~PIG represents the average abundance value for each transcripts when isolates PIG30/1 and BP-4 are combined. ***fc:*** *log*_2_ fold change of G10/1 vs BP~PIG comparison. FPKM values were used for calculations.

**Supplementary Table S2: Results from annotation procedure.** Table is organized in six columns as follows: **1**- ID assigned by our assembly protocol; **2-**ID of the corresponding cluster where the gen can be found; **3-** assigned ID corresponding to the two database models employed for the annotation procedure (PFAM, PANTHER); **4-** Description for the annotated sequence; **6-** Gene Ontology terms assigned by our annotation procedure.

**Supplementary Table S3:** **New hydrolases predicted from the transcriptomics assembly.** The file is by two sheets: **i)** Proteases, composed by 9 columns, **Transcript id:** ID assigned by cufflinks algorithm; Description: a concise description about the annotated hydrolase; columns 3 to 6 contains FPKM values for transcripts at each isolate analyzed in this work; column 7 contains log_2_ fold change values for comparison G10/1 vs BP~PIG; column 8 contains MEROPS annotation results. **ii)** EC annotation, composed of 4 columns, **Transcript id:** ID assigned by cufflinks algorithm; **GO IDs:** gene ontology terms; **GO Names:** description of gene ontology terms; **Enzyme Codes:** EC number assigned to transcript.

**Supplementary Table S4: Cluster Matrix.** Activity level of transcripts clusters used in this work.

**Supplementary Table S5: List of genes from *Tritrichomonas foetus* K1 reference genome with almost one predicted MYB binding site in its promoter.** For each gene is listed: Contig ID from draft assembly, nucleotide sequence ID (locus tag), Protein ID and functional annotation from reference genome (Description).

**Supplementary Table S6: List of principal bioinformatics tools used in this work.**

| **Analysis** | **Tool** |
| --- | --- |
| Quality evaluation of sequencing lectures | FASTQC^20^ |
| Adapter removal and quality filtering | trimmomatic^21^ |
| Short Reads alignment | hisat2^22^ |
| Transcriptomics assembly and quantification | Cufflinks^23^ |
| Assembly evaluation | CummeRbund^24^ |
| In silico translation | transeq^25^ |
| Transcriptome annotation | Interproscan^26^ |
| MEROPS database (v12.3) homology search | BLAST+^30^ |
| Enzyme Commission (EC) number mapping | blast2go^29^ |
| Sequence Alignment and visualization | JalView^34^ |
| Genomics region extraction | bedtools^39^ |


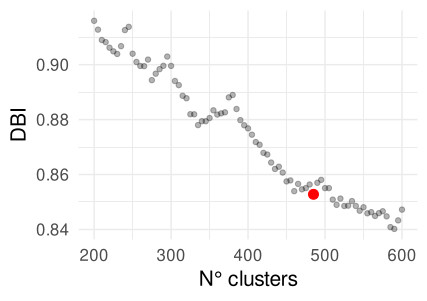
**Supplementary Figure S1:** **Davies-Bouldin index (DBI) as a function of the number of clusters.** The red point indicates the number of clusters used for further calculations.
